# Mange or Scabies in the Dog

**Published:** 1901-03

**Authors:** W. T. Campbell

**Affiliations:** Cincinnati, Ohio


### MANGE OR SCABIES IN THE DOG.

By W. T. Campbell, V.S.,
CINCINNATI, OHIO.

In introducing this subject I wish to point out that I have not selected it because I possess any claim to a special acquaintance with it, but rather because I consider it one of the highest importance to the practitioner.

While diseases of this particular class are not so dangerous to the animal's existence, yet we must place great importance on diseases of this nature, as they are the ones we are most often called upon to treat in canine practice.

All the diseases of the skin tend to produce bodily discomfort to the animal, and instead of eating it will be called upon to resist the attacks of the pests and will soon become very debilitated, and, if left alone, by the great reproduction of the pests the poor animal will be eaten alive, as you might say, by them.

Scabies is the most important of all the skin diseases, and is due to the presence of an animal parasite, which exists on the exudation which is developed by the irritation its presence causes on the skin.

It is characterized by more or less itching and eruptions of papules, vesicles, and pustules, which frequently coalesce. It is one of the oldest diseases known, and when fully developed it is one of the most tedious to treat.

Of this latter class I have had in the past few months a great many cases, and have made a few observations which may be of interest to some of the readers, as they have helped me and others who have also watched the cases. I shall give a history and the treatment of some of the cases, which were as follows:

About July 1st a fox terrier, high-bred, was brought to the hospital with a marked case of scabies, having been affected about a week. The animal was in a state of great excitement, the eyes and visible mucous membranes were reddened, the hair was matted, and great blotches had suppurated and were sloughing off. The skin was very hot and the animal was nearly mad from itching. The owner, who kept a large kennel, stated that he had been away and the attendant had become careless. When I stated the nature of the case to the owner he ordered me to inspect his kennel, in which, when I did, I found all his dogs, some twenty in number, more or less affected. Having such a large number to treat, I at once made preparation to treat them systematically, which I did in the following way:

Taking all the dogs out I made a big dip in a great trough I had, as follows: Creolin, one pound; zinci sulph. and plumbi acetate, one-half pound to twenty gallons of water.

The kennels were all whitewashed and disinfected and fresh litter placed in each box. After washing all the dogs in the dip I allowed them to dry, and after brushing them well with a soft brush I applied a solution of epicarin and sweet oil (which I believe, as others who have tried it, surpasses all other remedies in cases where itching is a prominent symptom).

Twice a day the oil and epicarin was applied all over the affected parts, and relief seemed immediate after the application, and I was surprised at the short space of time it took to heal them all.

Shortly afterward I was called to see a St. Bernard—the most pitiful sight I ever saw. He was sore from the head to the tail, and great masses of hair and skin had sloughed off. The poor animal had scratched himself raw and suffered intense pain.

I took him to the trough where I had treated the others, and gave him the same application and followed with the oil and epicarin, as before. Immediately after I had applied it he became easy and was sound asleep in a short while—the first time, the owner claimed, since he noticed the disease. I ordered the oil and epicarin used twice a day, and he improved rapidly, with the exception of a few places where the follicles had been destroyed, which healed but always remained bald.

In both these instances I had excellent results, and have had like results in a great many cases I have had under my care since, but before I was always having trouble with these cases because of the length of time it took to effect a cure. In all the cases I never saw the animal scratching after the first application of oil and epicarin, and before it was the scratching open of the healing wounds that kept the case standing so long.

I have inquired among my brother practitioners of my neighborhood and find this has always been their trouble, but upon trying the oil, as I advised them, they also met with the same success. I thought that by recording this I may find others who have the same trouble and who might profit by trying this method.

The dip need only be proportionate to the number of animals and the oil and epicarin be of equal parts, to which may be added ten parts of glycerin.

